# Recovery from borderline personality

**DOI:** 10.1192/j.eurpsy.2021.1348

**Published:** 2021-08-13

**Authors:** D. Duarte

**Affiliations:** Departamento De Psiquiatria E Saúde Mental, Centro Hospitalar Universitário do Algarve, Estoi, Faro, Portugal

**Keywords:** recovery, Borderline personality disorder

## Abstract

**Introduction:**

Recovery is much broader than experiencing remission of symptoms. It is understood as the experience of a subjectively significant and satisfying life, even if some symptoms of mental illness persist. The recovery process from borderline personality disorder (BPD) is complex and includes recognizing the need for change and developing greater self-acceptance.

**Objectives:**

Provide an overview of personal recovery from BPD.

**Methods:**

The authors did a non-systematic review in pubmed with the words: “borderline personality disorder” and “Recovery”.

**Results:**

BPD is a serious mental disorder characterized by a pattern of instability in interpersonal relationships, self-image and affections, marked by impulsiveness and (para) suicidal behaviors. Unemployment and difficulties in obtaining and maintaining employment are highly prevalent on BPD and add social exclusion and deterioration of physical and mental health. Recent long-term follow-up studies offer a optimistic scenario, indicating high rates of clinical remission (not equivalent to full recovery). Most psychotherapies, such as dialectical behavioral therapy (DBT) or mentalization-based therapy, have proven their effectiveness in treating emotional dysregulation, impulsivity and interpersonal difficulties. Teams working with people with BPD should develop comprehensive multidisciplinary care plans. The care plan should identify long-term goals that should be realistic, and linked to the short-term treatment aims and develop a crisis plan that identifies potential triggers that could lead to a crisis.

**Conclusions:**

Cognitive rehabilitation and psychoeducational interventions can be effective in individuals with BPD. These interventions can be easily implemented in mental health settings and offer benefits for improving overall functioning, which often remains affected after clinical remission in long-term follow-up.

